# Silicon- and Boron-Induced Physio-Biochemical Alteration and Organic Acid Regulation Mitigates Aluminum Phytotoxicity in Date Palm Seedlings

**DOI:** 10.3390/antiox11061063

**Published:** 2022-05-27

**Authors:** Saqib Bilal, Adil Khan, Muhammad Imran, Abdul Latif Khan, Sajjad Asaf, Ahmed Al-Rawahi, Masoud Sulaiman Abood Al-Azri, Ahmed Al-Harrasi, In-Jung Lee

**Affiliations:** 1 Natural & Medical Sciences Research Center, University of Nizwa, Nizwa 616, Oman; saqib@unizwa.edu.om (S.B.); sajjadasaf@unizwa.edu.om (S.A.); ahmed@unizwa.edu.om (A.A.-R.); 2 Institute of Genomics for Crop Abiotic Stress Tolerance, Department of Plant and Soil Science, Texas Tech University, Lubbock, TX 79409, USA; adilkhan@ttu.edu; 3 Division of Plant Biosciences, School of Applied Biosciences, College of Agriculture & Life Science, Kyungpook National University, 80 Dahak-ro, Buk-gu, Daegu 41566, Korea; m.imran@knu.ac.kr; 4 Department of Engineering Technology, University of Houston, Sugar Land, TX 77479, USA; 5 Ministry of Agriculture, Fisheries and Water Resources, Muscat 100, Oman; masoud.alazri@maf.gov.om

**Keywords:** date palm (*Phoenix dactylifera* L.), silicon, boron, aluminum, oxidative stress, organic acid

## Abstract

The current study aimed to understand the synergistic impacts of silicon (Si; 1.0 mM) and boron (B; 10 µM) application on modulating physio-molecular responses of date palm to mitigate aluminum (Al^3+^; 2.0 mM) toxicity. Results revealed that compared to sole Si and B treatments, a combined application significantly improved plant growth, biomass, and photosynthetic pigments during Al toxicity. Interestingly, Si and B resulted in significantly higher exudation of organic acid (malic acids, citric acids, and acetic acid) in the plant’s rhizosphere. This is also correlated with the reduced accumulation and translocation of Al in roots (60%) and shoots (56%) in Si and B treatments during Al toxicity compared to in sole Al^3+^ treatment. The activation of organic acids by combined Si + B application has significantly regulated the *ALMT1*, *ALMT2* and plasma membrane ATPase; *PMMA1* and *PMMA3* in roots and shoots. Further, the Si-related transporter *Lsi2* gene was upregulated by Si + B application under Al toxicity. This was also validated by the higher uptake and translocation of Si in plants. Al-induced oxidative stress was significantly counteracted by exhibiting lower malondialdehyde and superoxide production in Si + B treatments. Experiencing less oxidative stress was evident from upregulation of *CAT* and *Cyt-Cu/Zn SOD* expression; hence, enzymatic activities such as polyphenol oxidase, catalase, peroxidase, and ascorbate peroxidase were significantly activated. In the case of endogenous phytohormones, Si + B application demonstrated the downregulation of the abscisic acid (ABA; *NCED1* and *NCED6)* and salicylic acid (SA; *PYL4*, *PYR1*) biosynthesis-related genes. Consequently, we also noticed a lower accumulation of ABA and rising SA levels under Al-stress. The current findings illustrate that the synergistic Si + B application could be an effective strategy for date palm growth and productivity against Al stress and could be further extended in field trails in Al-contaminated fields.

## 1. Introduction

After silicon and oxygen, aluminum (Al) is considered as one of the most abundant elements on the earth’s crust, occurring in the form of silicates or other deposits [1,2]. The accumulation of Al in acidic soil (with a pH of 5.5 or lower) converts it into a phytotoxic form of Al^3+^_,_ which negatively influences the plant root system and subsequently ruins the aerial parts of plants due to immediate root deterioration [3,4,5,6]. The toxicity of Al^3+^ in roots inhibits cell division and elongation, causing root apices to be swollen and leading to poor or no root-hair development, oxidative damage, and the impairment of several pathways responsible for signal transduction [7,8,9]. In parallel, the higher accumulation of Al^3+^ is also a major threat to humans and animals through the food chain [10]. For a higher rate of aluminum transportation from soil to plants and the food chain, there is an urgent need to control the higher uptake of aluminum in edible crops, which will lower possible health risks and problems in humans.

In recent decades, various mechanisms have been discussed, and employed by plants, to overcome Al^3+^ toxicity, for instance, the chelation of Al^3+^ by different organic acids such as citrate, oxalate, and malate, or via neutralizing the absorbed Al^3+^ inside the root and shoot symplasm. Moreover, plant roots secrete organic acids in response to Al^3+^ stress, which chelates Al^3+^ in the rhizosphere, protecting roots. The exudation of the organic acid is usually regulated by membrane-localized organic acid transporters such as the Al-activated malate transporter and multidrug and toxic compound extrusion [11]. However, several studies have described the regulation of organic acids by different exogenous signaling molecules and phytohormones such as abscisic acid (ABA), indole acetic acid (IAA), salicylic acid (SA), ethylene (ET), and nitric oxide (NO) [12,13,14]. Moreover, overcoming Al^3+^ toxicity, various proteins, phenolic compounds, and tannins have been reported that make complexes with the Al^3+^, which subsequently results in the compartmentalization of the Al^3+^ inside the vacuoles and thereby reduces Al^3+^ toxicity but leads to higher accumulation [15,16]. Various studies demonstrated the higher production of ROS in plants exposed to Al^3+^ stress, which subsequently induced oxidative stress, caused the peroxidation of the cell membrane, negatively influenced the structural integrity of cells, caused chromosomal abnormality, and led to cell death [17,18]. Furthermore, Al^3+^ toxicity is known for hampering the uptake and metabolism of different nutrients, such as Cu, N, Ca, Mn, Mg, P, and Fe [19]. It poses a higher affinity for reacting with the negatively charged plasma membrane.

The presence of silicon (Si) in the soil is considered a beneficial mineral element for plant growth and development and for mitigating biotic and abiotic stress conditions. Therefore, the stress-alleviating effects of Si vary with plant species, and generally a significant stress-mitigating role is reported in plants with a higher accumulation of Si in different tissues [20]. Similarly, Si enhances Al^3+^ tolerance in plants by the activation of antioxidant activities [21,22], the formation of the Si-Al^3+^ complex [23], pH increase [24], the enhancement of organic acid anions, mineral uptake, and the exudation of phenolic compounds [25], as well as hormonal signaling [21,26,27,28]. While Si’s involvement in ameliorating Al-induced stress could be due to altering Al transporter activities, conclusive evidence regarding the effect of Si on regulating Al transporters is not available. Moreover, the formation of complexes of boron and silicon in soil under certain environmental conditions due to sharing considerable chemical similarity has been reported to alter heavy metal phytotoxic effects, uptake, and transportation [25]. However, there is also a shortage of reports regarding the stress-alleviating role of Si under Al^3+^ toxicity in the presence of boron in date palm.

Boron has been placed on the list of essential micronutrients and is considered an essential element for plant growth and the development of higher crop plants [18,29,30,31]. The deficiency of B in plants leads to many deformities in plants, such as root growth inhibition; the induction of chlorotic disorders in leaves; a reduction in yield; and the impairment of the cellular components, structure, and composition of the cell wall [31,32,33]. Similarly, the alleviation of Al3+-induced stress by the exogenous treatment of B in different plants has been reported previously by many researchers in different plants [29,34,35,36,37,38,39,40,41,42,43,44,45,46,47,48,49,50,51,52,53,54,55]. Moreover, these studies have suggested that B possibly overcomes the consequences of Al^3+^ toxicity by restricting Al binding sites. B is reported to enhance alkalization in pea roots and thereby reduce Al toxicity [5], whereas high rainfall may wash out the alkalinity and alkalinity-related earth elements from the soil, thus causing acidic conditions, the accumulation of Al contamination, and B deficiency problems [29].

In Oman, date palm (*Phoenix dactylifera*) is an important fruit crop and makes Oman the eighth largest producer globally, with an average of 260,000 mt per annum production. Date palm is reported to be grown in 50% of Oman’s cultivated land and comprises 82% of all the cultivated fruit crops in Oman [36,37]. Metals contamination due to rapid urbanization and anthropogenic activities can negatively affect date palm growth and the quality of fruit [38,39]. During germination and the initial growth stages, date palm plants are reported to be fragile and tender and to adopt different sensing mechanisms to achieve germination under favorable environmental conditions [39]. Hence, the germination and early growth stages of date palm are adversely impacted by heavy metal pollution. Previously, Awad et al. [40] reported that the toxic effect of Al significantly inhibited the embryo production of date palm callus and negatively affected biochemical activities, including soluble carbohydrates, total proteins, antioxidative activities, free amino acids, and molecular responses. The adverse impacts of aluminum toxicity on date palm seedlings growing in Al-contaminated soil at biochemical and molecular levels have not been explored. Limited numbers of scientific reports provide enough information regarding the interaction of Si and B for influencing Al toxicity in date palm. Therefore, the current study aimed to unveil the ameliorative effect of Si and B and their combination on the Al-induced inhibition of growth attributes, and oxidative damage at physiological, biochemical, and molecular levels. The synergism of Si and B under Al-toxicity in date palm was elucidated by stress-related hormonal modulation, ROS-homeostasis, and stress-responsive gene alteration. Moreover, the effect of Si and B on modulating organic acid exudation and their subsequent impact on Al-uptake and Al-translocation from roots to shoots and their responsive genes expression were also explored.

## 2. Material and Methods

### 2.1. Plant Growth and Experimental Design

Oman Botanical Garden provided seedlings of date palm (Khalas cultivar). Uniform seedlings in length and number of leaves were transplanted into pots containing sphagnum peat moss (electrical conductivity (EC) 2.0 (dS m^−1^), moisture content (38.5%), pH 4.5–5.5, organic matter 91.1% (sodium chloride 850 mg kg^−1^, nitrogen 800–2500 mg kg^−1^, and phosphorus 150–850 mg kg^−1^). The seedlings were treated with distilled water (50 mL to each pot) in the growth chamber for three months to equilibrate the seedlings growth. The growth chamber conditions were adjusted to 12 h light (30 °C; relative humidity 60%) and 12 h dark (25 °C; humidity 60 %) conditions. Thereafter, date palm seedlings were subjected to Si (Na_2_SiO_3_; 1.0 mM), B (H_3_BO_3_; 10 µM), and Al^3+^ (AlCl_3_; 2.0 mM). Si, B, and Al^3+^ concentrations were selected through a pre-experiment on various plants [29,41,42,43]. For the experiment, date palm seedlings were treated with 50 mL solution four times a week. Seedlings were subjected to one of eight treatments: (1) Ct (without Si, B and Al^3+^), (2) Si (1.0 mM), (3) B (20 µM), (4) Si + B (1mM of Si; 20 µM B), (5) Al^3+^ (2.0 mM), (6) Si + Al^3+^ (1.0 mM of Si; 2.0 mM of Al^3+^), (7) B + Al^3+^ (10 µm of B; 2.0 mM of Al^3+^), and (8) Si + B + Al^3+^ (1.0 mM of Si, 10 µm of B, and 2.0 mM Al^3+^). All the solutions were prepared in distilled water. The pH of all nutritional solutions was adjusted to 6.0. The pots were put in the greenhouse in a randomized arrangement with five replications. For 10 weeks, 50 mL of Si, B, and Al^3+^ was administered simultaneously to the plant’s root zone. After 10 weeks of treatment, the growth characteristics were recorded and plants were collected in liquid nitrogen and kept at −80 °C. The experiment was done twice, each time with 10 replications.

### 2.2. Chlorophyll a and Chlorophyll b

Leaf samples (200 mg) of date palm seedlings were ground in mortar using 80% acetone to analyze photosynthetic pigments (Chl a, Chl b, and carotenoids). To estimate the Chl a and Chl b content, the methodology of Sumanta et al. [44] was employed. The absorbance values for Chl a, Chl b, and carotenoids were recorded at 663, 645, and 470 nm for Chl a, Chl b, and carotenoid, respectively.

### 2.3. Quantification of Malondialdehyde (MDA)

The extent of lipid peroxidation or formation of MDA was estimated using the methodology reported by Bilal et al. [45] and Khan et al. [46]. Briefly, 10 mM phosphate buffer extracted tissue homogenates (pH 7.0). In a reaction tube, 0.2 mL of tissue homogenate was mixed with 0.2 mL of 8.1 percent sodium dodecyl sulfate (SDS), 1.5 mL of 20 percent acetic acid (pH 3.5), and 1.5 mL of 0.81 percent thiobarbituric aqueous acid (TBA) solution for the measurement of MDA. After that, the mixture was heated for 60 min in boiling water followed by cooling to room temperature and the addition of 5.0 mL butanol:pyridine (15:1 *v/v*) solution. A spectrophotometer was used to measure the optical density of the resultant pink solution at 532 nm after the top organic layer was removed.

### 2.4. Determination of Superoxide (O_2_^•−^)

The measurements of O_2_^•−^ were done according to the method reported by Gajewska and Skłodowska [47] and Khan et al. [42]. Briefly, 1.0 g of fresh plant material was immersed in phosphate buffer (pH 7.0) containing sodium phosphate (10 mM), nitrobluetetrazolium (NBT) (0.05 percent; *w/v*), and sodium azide (NaN_3_), to prepare the homogenate for the reaction (10 mM). For 1 h, the mixture was left at room temperature. Afterward, 5 mL of the mixture was put in a new tube and heated at 85 °C for fifteen min. The mixture was then chilled and vacuum filtered. A spectrophotometer was employed to determine the sample’s absorbance at 580 nm. The experiment was appropriately conducted three times.

### 2.5. Antioxidant Enzyme Assay

The antioxidant enzymes ascorbate peroxidase (APX), polyphenol oxidase (PPO), peroxidase (POD), and catalase (CAT) were analyzed as described by Bilal et al. [45], with minor modifications. Briefly, date palm leaves and roots (100 mg) were crushed via liquid nitrogen. Phosphate buffer (100 mM) was added to the sample to obtain a homogenous pH 7.0. The resulting homogenate was centrifuged for 30 min at 10,000 rpm and 4 °C L.

The reaction mixture for POD analysis was made up of 0.1 M potassium phosphate buffer (pH 6.8), 50 µL H_2_O_2_ (50 M), 50 µL pyrogallol (50 M), and 100 µL crude extract sample. This mixture was incubated at 25 °C for 5 min; then, 5% H_2_SO_4_ (*v/v*) was added to cease the enzymatic activity. An optical density of 420 nm was used to determine the quantity of purpurogallin produced. The reaction mixture for determining polyphenol oxidase (PPO) activity used comparable components to the POD assay; however, without H_2_O_2_, the resultant activity was measured at 420 nm. An increase of 0.1 units of absorbance was used to quantify a single unit of PPO and POD. The CAT activity was investigated as described by Aebi [48]. Briefly, the crude enzyme extract was added to 0.2 M H_2_O_2_ in 10 mM phosphate buffer (pH 7.0), after which the CAT activity was determined as a decrease in absorbance at 240 nm and expressed as units (one unit of CAT was defined as the ng of H_2_O_2_ released/mg protein/min).

For the quantification of APX, 1 mL phosphate buffer (50 mM; pH 7.0) containing 1.0 mM ascorbic acid and 1.0 mM EDTA was used for extraction, followed by homogenization at 50 Hz for 30 s and centrifugation at 4830× *g* at 4 °C for 15 min. Subsequently, the supernatant was added into the phosphate buffer solution (pH 7.0) having 15 mM AsA and 0.3 mM H_2_O_2_. At 290 nm, the reaction mixture was examined. A variable quantity of absorbance at 290 nm per minute was defined as one unit of APX. The enzymatic tests were performed three times, with three replications each time.

### 2.6. Micronutrient Quantification

Silicon quantification was investigated by the method described by Ali et al. [49] via ICP-MS (Optima 7900DV, Perkin-Elmer, Waltham, MA, USA) by using freeze-dried date palm roots and leaves (0.05 g sample). The experiment was repeated three times, and each time comprised of three replications.

### 2.7. Determination of Organic Acids

Organic acids in seeds were extracted according to the method previously described by Bilal et al. [50]. Briefly, a rotary evaporator was used for evaporating the extracted samples, followed by re-dissolving the dried residues in deionized water and filtering through a 0.45-μm filter. Furthermore, HPLC (Millipore Corp., Waters Chromatography, Milford, MA, USA) was used for quantifying organic acids in samples. Different organic acids such as citric acid, malic acid, succinic acid, and acetic acids were measured by comparing their peak values to the peak values of their respective standards.

### 2.8. Abscisic Acid and Salicylic Acid Extraction and Quantification

Endogenous abscisic acid (ABA) was extracted and quantified according to the modified protocol as described in Shahzad et al. [51] and Khan et al. [46]. Salicylic acid was extracted and quantified from freeze-dried samples according to Seskar et al. [52] as described by Bilal et al. [53]. For detail, see Supplementary File S1.

### 2.9. Gene Expression Analysis

RNA was extracted from date palm leaves using an extraction buffer (0.25 M, NaCl; 0.05M, Tris-HCl (pH = 7.5); 20 mM, EDTA; 1% (*w/v*) SDS; 4% polyvinylpyrrolidone (*w/v*)) as reported by [41]. Briefly, 750 µL of the extraction buffer and chloroform: isoamyl alcohol (CI; 24:1 *v/v*) was mixed with a 2-mL RNase-free microcentrifuge tube followed by the addition of 40 µL β-mercaptoethanol. After that, a fine powder (100 mg) of the sample was carefully transferred to a 2 mL tube containing the extraction buffer and CI. Subsequently, the mixture was vortexed, followed by incubation for 15 min at 20 °C and 10 min of centrifugation (12,000× *g*) at 4.0 °C. Then, supernatant (600 µL) was transferred to a new tube, followed by the addition of the same volume of CI. The gently mixed solution was centrifuged (12,000× *g*) for 10 min at 4.0 °C. The upper layer was transferred to a new tube and 3 M sodium acetate (pH = 5.2) of 1/10 volume was added. Absolute ethanol was added for precipitation, followed by gentle mixing and incubation for 45 min at 4.0 °C. After that, centrifugation of the sample was carried out at 4.0°C for 10 min. The pellet was collected, and diethyl pyrocarbonate-treated water (200 µL) was added, followed by the addition of 500 µL of 10 M LiCl, and kept on ice for 60 min. Sample was centrifuged at 4.0 °C for 10 min at 12,000× *g*, pellet was collected and washed with 70% ethanol.

The synthesized cDNA was used to amplify genes (Table S1). A total of 11 genes’ expression was assessed in each sample related to Si and Al^3+^ transport, the ABA biosynthesis pathway, and oxidative stress regulation. *Actin* gene was used as a reference for all the primers. Power up “SYBR” green Master Mix was used for the thermo-cycler (Quantstudio 5 by applied biosystems life technologies) PCR reaction. The reaction was performed at a specific condition such as 94 °C for 10 min in stage 1; 35 cycles of PCR reaction at 94 °C for 45 s; 65 °C for 45 s; and 72 °C for 1 min; finally, the extension temperature was set at 72 °C for 10 min. The threshold level of 0.1 was set for gene amplifications. The experiment was repeated three times and each time was comprised of three replications.

### 2.10. Statistical Analysis

GraphPad Prism was used to create all graphs and data analysis (v8.01; San Diego, CA, USA). All values are expressed as mean ± standard deviation. Mean values were analyzed using Duncan’s multiple range tests with a significant difference among treatments by ANOVA using SAS software (V9.1, Cary, NC, USA) to reveal significant to non-significant treatments by maintaining *p* < 0.05 and was represented by different lower-case alphabets.

## 3. Results

### 3.1. Effects of Si, B, and Their Combination on Date Palm Growth Parameters under Al^3+^ Phytotoxicity

**Figure 1:**
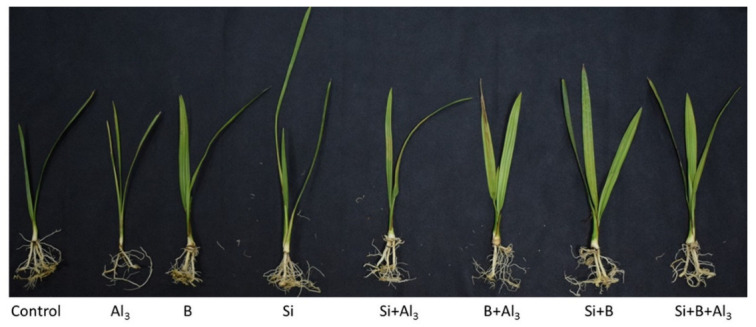
Application of sole silicon (Si) and Boron (B) and their integrative effects on date palm growth under Al stress.

As shown in Table 1 and Figure 1, in the absence of Al^3^^+^ stress, sole Si and B, as well as their combination (Si + B), substantially improved the shoot length by approximately 40%, 33%, and 55%, respectively, and shoot dry weight by 52%, 41%, and 78%, respectively, as compared to the non-treated plants. Similarly, significant enhancement was detected in the root length under sole Si, B, and their combination (Si + B) by approximately 27%, 31%, and 45% and root dry weight by 21%, 14%, and 25%, respectively, relative to non-treated plants in the absence of Al^3^^+^ stress. However, exposure to Al^3+^ toxicity significantly reduced the shoot length and root length of the control, Si, B, and Si + B treatments by 23%, 13%, 15%, and 16%, respectively, and root length by 25%, 8%, 7%, and 4%, respectively, compared to their treatment of control conditions, while in comparison with the respective treatment of no-stress conditions, control (Ct)-, Si-, B-, and Si + B-treated plants under Al^3+^ stress illustrated decreased shoot dry weight by nearly 28%, 23%, and 15% and root dry weight by 31%, 21%, 26%, and 24%, respectively. However, the application of Si and B and their combination (Si + B) considerably ameliorated Al3+-induced damage to growth attributes compared to non-treated plants, e.g., sole Si, B, and their combination demonstrated significant enhancement in shoot length compared with only Al^3+^-treated plants by approximately 56%, 46%, and 72% and root length by 57%, 65%, and 87%, respectively (Table 1). On the other hand, the shoot and dry weight of sole and combined Si- and B-treated plants under Al^3+^ toxicity also presented a statistically significant improvement compared to only Al3+-treated date palm seedlings.

Similarly, date palm seedlings treated with either Si, B, or their combination maintain chlorophyll pigments such as Chl *a*, Chl *b*, and carotenoids. Under control conditions, the application of Si, B, and their combination (Si + B) did not present a statistically significant alteration in Chl *a* level in leaves compared to non-treated plants. On the contrary, an increase was observed in Chl *b* level by the interaction of Si + B by 91%, which was statistically equally followed by sole Si- and B-treated plants compared to control plants under no-stress conditions, whereas under Al^3+^ treatment, combined Si + B and sole Si application revealed a significantly higher Chl *b*, while only Al^3+^ was detected with the lowest level of Chl *b.* In the case of carotenoids under no-stress conditions, the combination of Si and B presented a significantly higher level, followed by sole B, Si, and non-treated control plants. Not surprisingly, Al^3+^ treatment altered the carotenoids level in date palm seedlings, but the supplementation of exogenous Si, B, and their combination considerably improved the level by approximately 66%, 85%, and 144%, respectively, compared to only Al3+-treated date palm seedlings (Table 1). To conclude, Si, B, and their combination (Si + B) showed a synergistic effect against Al3+-induced chlorosis in date palm seedlings.

### 3.2. Accumulation and Translocation of Si, B, and Al^3+^ in Date Palm

The accumulation of B, Al^3+,^ and Si and their translocation from the roots to the shoots of date palm were investigated via ICP-MS (Table 2). The accumulation of Al^3+^ was considerably higher in the roots than the shoots. In the absence of Al^3+^ stress, combined Si + B-treated plants exhibited a higher uptake of Al^3+^ in shoots, followed by the control plants and the sole B- and Si-treated plants, respectively. However, combined Si and B application significantly reduced Al^3+^ accumulation in the roots compared to only Al3+-treated plants by approximately 59%, followed by sole B- and Si-treated plants by 49% and 40%, respectively. Similarly, Al^3+^ uptake was significantly reduced in shoots of combined Si + B plants by 2.3, 1.3, and 1.6 fold compared to non-treated, sole Si-, and B-treated plants, respectively, under Al^3+^ stress.

Similarly, a significantly higher amount of Si was determined in both the leaves and roots of the date palm seedling with the combined and sole application of exogenous Si and B compared to control plants. Interestingly, the application of exogenous Al^3+^ inhibited the uptake of Si in roots and shoots compared to non-treated plants of control conditions by 34% and 19%, respectively (Table 2), while under Al^3+^ toxicity, the combined and sole application of Si and B significantly enhanced the uptake of Si in roots and shoots compared to only Al^3+^ plants by (65%, 86%, and 44%) and (68%, 84%, and 30%), respectively. Interestingly, Al^3+^ toxicity did not alter B uptake significantly in the roots and shoots of only Al3+-treated plants compared to control plants. However, exogenous B and combined Si + B under Al^3+^ stress exhibited the equally maximum accumulation of B in roots and the sole application of B treatment presented higher accumulation in shoots.

### 3.3. Effect of Exogenous Si, B, and Their Interaction on Lipid Peroxidation and Superoxide Anion Accumulation under Al^3+^ Stress

**Figure 2:**
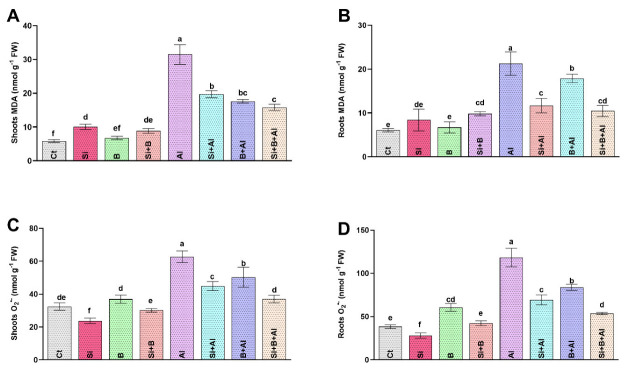
The effect of sole silicon (Si), boron (B), and their combination on (**A**,**B**) lipid peroxidation (malondialdehyde, MDA) and (**C**,**D**) superoxide anion (O_2_^•^^−^) modulation under Al-induced toxicity in date palm roots and shoots. Bars with different letters describe statistically significant differences (*p <* 0.05) between means by using one-way analysis of variance (ANOVA), followed by Duncan’s multiple range test (DMRT). Bars describe mean (of four replicates) ± standard error.

The ROS-induced peroxidation of lipid membranes and superoxide anion (O_2_^•−^) produce stress-induced damage at the cellular level. We found that Al^3+^ stress significantly increased the production of MDA and O_2_^•−,^ which damage the cell structure due to its highly reactive and toxic nature. A significant augmentation in MDA level was noticed only in the roots of Al3+-treated plants compared to the plants treated with Si, B, and their combination (Figure 2). The interaction of Si and B significantly mitigated the toxic effects of Al^3+^ toxicity. It exhibited the lowest level of MDA in roots by approximately 50%, followed by sole Si- and B-treated plants with 45% and 16% reduced levels, respectively, compared to only Al3+-treated plants. Likewise, the MDA level in the shoots was either not significantly or less altered by Si, B, and Si + B in the absence of Al^3+^ stress. However, exposure to Al^3+^ significantly upregulated MDA levels in only Al3+-treated plants by 37%, 43%, and 50% compared with Si, B, and their combination, respectively.

Moreover, in the absence of Al^3+^ stress, the application of sole Si revealed a significantly reduced level of O_2_^•−^ in both shoots and roots compared to other treatments. Unlike the absence of Al^3+^ stress, the O_2_^•−^ level was significantly elevated under Al^3+^ stress in both roots and shoots. The alleviated level of O_2_^•−^ was significantly inhibited under the interaction of Si + B by demonstrating approximately 55%, 23%, and 36% lower accumulation in roots and 40%, 17%, and 26% reduced level in shoots, in comparison with only Al3+-treated, Si-treated, and B-treated plants, respectively.

To conclude, Si, B, or Si + B reduced the MDA and O_2_^•−^ level more significantly under Al^3+^ stress than the absence of Al^3+^ stress.

### 3.4. Modulation of Antioxidant Activities by Si, B, and Their Interaction

**Figure 3:**
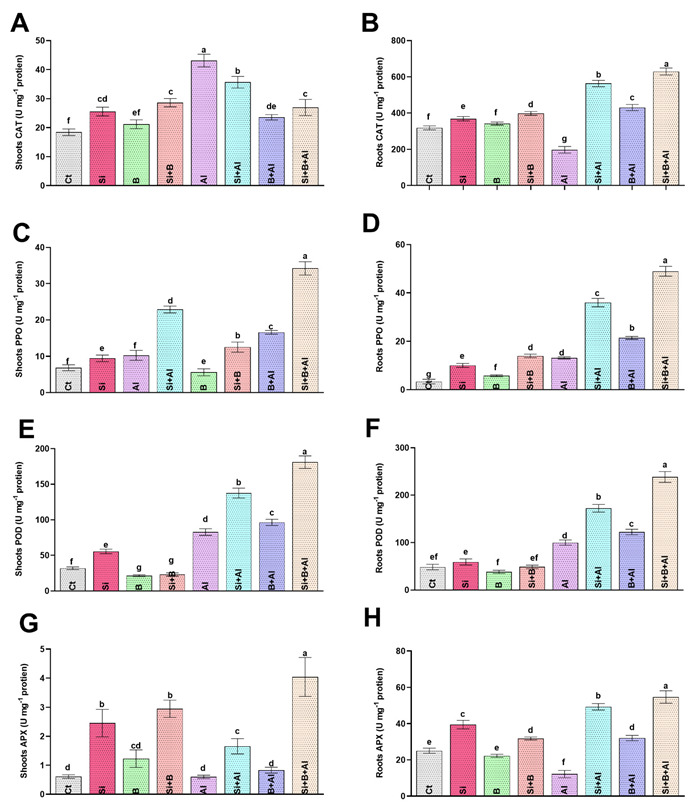
The effect of sole silicon (Si), boron (B), and their combination on antioxidant regulation (catalase, CAT; polyphenol oxidase; PPO peroxidase, POD; and ascorbate peroxidase, APX;) of date palm roots and shoots under Al-induced stress. (**A**,**B**) CAT, (**C**,**D**) PPO, (**E**,**F**) POD, (**G**,**H**) APX. Bars with different letters are describing statistically significant differences (*p* < 0.05) between means by using one-way analysis of variance (ANOVA), followed by Duncan’s multiple range test (DMRT). Values describe the mean (of four replicates) ± the standard error.

Metals toxicity is known for hampering the plant antioxidative system due to excess oxidative stress. Therefore, Si, B, and Si + B interaction were evaluated by analyzing antioxidant enzyme activities (CAT, PPO, POD, and APX) under Al^3+^ stress in date palm seedlings (Figure 3). In the absence of Al^3+^ stress, the control treatment exhibited the lowest CAT level in shoots, while Si + B treatment illustrated a significantly enhanced level compared to other treatments. Under the exposure to Al^3+^ toxicity, sole Al^3+^-treated plants demonstrated approximately 134%, 21%, 83%, and 60% increase in the CAT level of shoots compared to control, sole Si, B, and their combination, respectively. On the contrary, the CAT level of roots in the absence and presence of Al^3+^ stress was significantly elevated compared to other treatments. Al^3+^ toxicity significantly reduced CAT in the roots of only Al3+-treated plants by approximately 2.8-, 2.1-, and 3.1-fold compared to the sole Si, B, and their combination, respectively. Likewise, Si + B slightly increased the activity of PPO in shoots and roots compared to the rest of the treatments under normal conditions. In the case of Al^3+^ stress, the PPO level was significantly enhanced in all treatments compared to normal conditions. A significantly enhanced PPO level was detected under Al^3+^ stress in combined Si + B treatment by approximately 3.3-, 1.5-, 2.0-fold in shoots and 3.7-, 1.3- and 2.2-fold in roots compared with Al^3+^-treated, sole Si-, and B-treated plants, respectively.

In terms of POD regulation, the sole B and Si + B combination presented significantly decreased POD levels in shoots equally, while significantly reduced POD levels in roots were detected in sole B-treated plants compared to other treatments under normal conditions (Figure 2). However, in response to Al3+-induced ROS, a significant elevation in POD level was observed in roots and shoots compared to normal conditions. The combination of Si + B markedly enhanced the POD level in shoots and roots under Al^3+^ stress by 2.1- and 2.3-fold, followed by sole Si-treatment with 1.6- and 1.7- fold and sole B-treatments with 1.1- and 1.2-fold, as compared to sole Al^3+^ treatment, respectively. Likewise, the level of APX was markedly enhanced in the roots compared to the shoots of date palm seedlings. Under normal conditions, sole Si-treated plants illustrated a significantly enhanced level of APX in roots compared to other treatments. However, the APX activity of the roots was significantly inhibited in sole Al3+-treated plants compared to control plants. However, the Al^3+^ toxicity in roots was substantially mitigated by the combined application of Si + B-treated plants via demonstrating approximately 4.6-, 1.1-, and 1.7-fold higher levels compared to sole Al^3+^, Si-, and B-treated plants, respectively. A slight variation was noted in the APX level of shoots between normal and Al^3+^ stress conditions. Nevertheless, the combined treatment of Si + B presented a significantly higher content of APX in shoots in normal conditions, as well as Al^3+^ stress, in comparison with other treatments.

To conclude, Si, B, and their combination boost Al^3+^ stress tolerance in date palm seedlings by upregulating the antioxidant enzymes, which help date palm seedlings experience less oxidative stress under normal and Al^3+^ stress conditions.

### 3.5. Effects of Si, B, and Si + B on the Endogenous ABA and SA Levels in Date Palm Seedlings under Normal and Al^3^^+^ Stress Conditions

The phytohormones act as signaling molecules under abiotic stresses by initiating different signal transduction pathways to cope with the adverse effect. In the current study, we determine the endogenous ABA and SA levels in all the treatments to understand the crosstalk of exogenous Si and B and their combination with endogenous ABA and SA under normal and Al^3+^ stress conditions.

**Figure 4:**
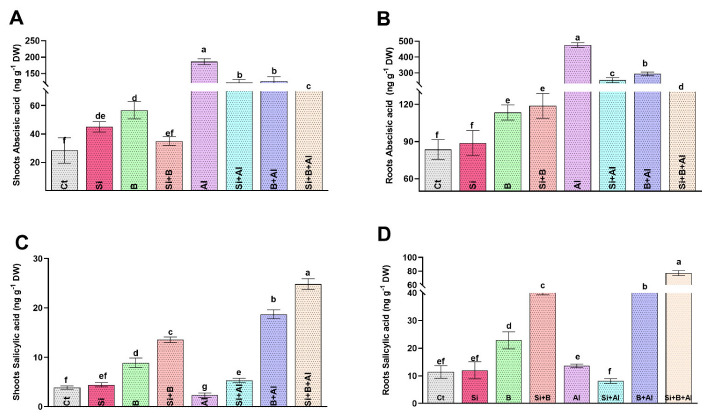
Endogenous hormonal (abscisic acid and salicylic acid) regulation by the sole and interactive effects of Si and B administration under Al-induced stress in date palm shoots and roots. (**A**,**B**) Abscisic acid, (**C**,**D**) Salicylic acid. The bars illustrate means (of four replications) ± standard error. The means with different letters are significantly different (*p* < 0.05) as evaluated by the Duncan Multiple Range Test (DMRT).

In the current study, the ABA level was increased significantly (around 6.5-fold) in the shoot of Al^3^^+^-treated seedlings compared to control plants. The escalated level of ABA in date palm leaves under Al^3+^ stress was significantly reduced by applying sole Si, B, and their combination (Figure 4). For instance, the implementation of Si, B, and Si + B decreased the level of ABA under Al^3+^ stress conditions by approximately 34%, 32%, and 44%, respectively, relative to sole Al^3+^-treated seedlings. Likewise, exposure to Al^3+^ toxicity enhanced the ABA level significantly by approximately 5.8-fold in the root of sole Al^3+^-treated seedlings compared to control plants. Interestingly, the escalated level of ABA under Al^3+^ stress in roots decreased significantly upon supplementation of Si, B, and their combination by approximately 1.8-, 1.6-, and 3.0-fold, respectively, compared to sole Al^3+^-treated plants (Figure 4B).

Similarly, the SA levels in the shoots of sole Al^3+^-treated seedlings decreased by approximately 39% compared to the control seedlings (Figure 4C). On the contrary, a significant enhancement in the level of shoots SA was observed upon the supplementation of Si, B, and their combination under Al^3+^ stress by approximately 56%, 87%, and 90%, respectively, compared with the sole Al^3+^ plants, while in the roots SA level, no significant changes were noticed between the control and sole Al^3+^ treated plants. However, the combination of Si + B followed by sole B and Si significantly exhibited an increased level of SA, respectively, compared to the sole Al3+-treated plants.

### 3.6. Effect of Exogenous Si and B on the Regulation of Citric Acid, Malic Acid, Succinic Acid, and Acetic Acid under Al^3+^ Stress Conditions

**Figure 5:**
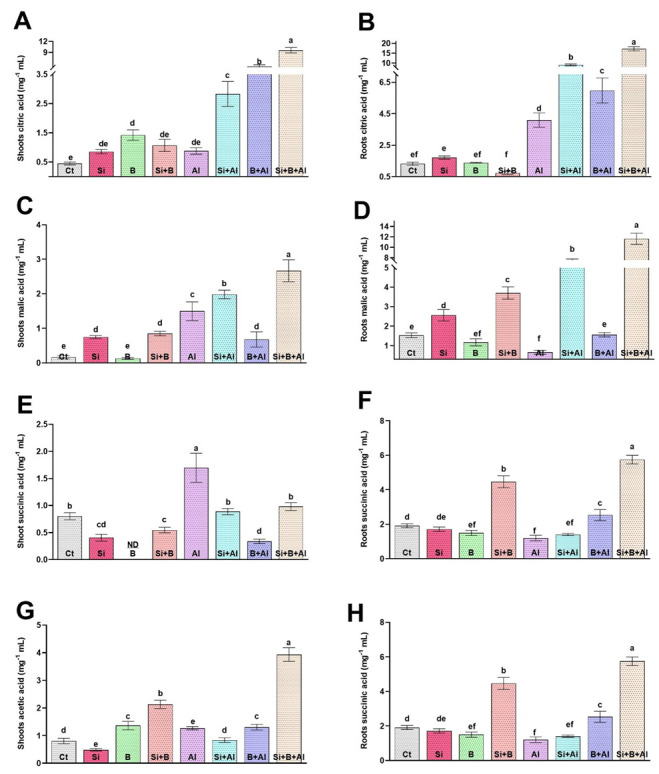
The sole and in-combination effect of Si and B on the organic acid regulation of date palm shoots and roots under Al-induced stress. (**A**,**B**) Citric acid, (**C**,**D**) Malic acid, (**E**,**F**) Succinic acid, (**G**,**H**) APX Bars with different letters are describing statistically significant differences (*p <* 0.05) between means by using one-way analysis of variance (ANOVA), followed by Duncan’s multiple range test (DMRT). The bars describe the mean (of four replicates) ± standard error.

Under Al^3+^ stress, plants adopt various mechanisms to overcome Al^3+^ toxicity, either preventing Al^3+^ from root due to the chelating of Al^3+^ by organic acid (such as citrate, oxalate, and malate) by neutralizing the absorbed Al^3+^ inside the root and shoot symplasm. The production of organic acids such as citric acid, malic acid, succinic acid, and acetic acid under Al^3+^ stress conditions is known for its vital role in detoxifying Al^3+^ toxicity. We found that under Al^3+^ Stress, citric acid increased significantly as compared to non-Al3+-treated seedlings (Figure 5). Interestingly, a low level of citric acid was detected in shoots under the absence of Al^3+^ stress in all treatments. However, applying a combination of Si + B significantly enhanced the citric acid level in the shoots under Al^3+^ stress, followed by sole B-treated and Si-treated plants, respectively, compared with only Al3+-treated plants. In comparison with shoot, the level of citric acid was significantly enhanced in roots by combining Si + B under Al^3+^ stress. Sole Al^3+^ treated plants exhibited approximately 4.2-, 2.2-, 1.4-fold lower levels of citric acid in roots compared to the plants treated with combined Si + B and sole Si- and B-treated plants, respectively. A similar trend was detected in the case of malic acid production in shoots and roots under Al^3+^ stress, and the production of malic acid in roots was detected to be higher than in the shoots. For example, the combined application of Si + B illustrated approximately 19.3- and 1.8-fold higher levels of citric acid in roots and shoots under Al^3+^ stress compared with sole Al^3+^ plants, respectively. Likewise, sole and combination Si and B exhibited a very low or insignificant effect compared to control plants on the succinic acid level of shoots and roots in the absence of Al^3+^ stress, while sole Al^3+^ treated plants presented significantly higher succinic acid production in both shoots and roots, followed by sole Si-treated and combined Si + B-treated plants, respectively.

On the contrary, the acetic acid level was significantly enhanced by the combined treatment of Si + B in both roots and shoots under control and stress conditions, compared to other treatments. The exposure of Al^3+^ toxicity significantly reduced the acetic acid level in the roots of sole Al3+-treated plants compared to their respective control plants by 1.7-fold, whereas Si + B-treated plants demonstrated approximately 5.1- and 3.2-fold greater levels of succinic acid in comparison with the roots and shoots of sole Al^3+^ treated plants, respectively.

### 3.7. Expression of Genes Related to Si and Al^3+^ Transport, ATPase H^+^ Pump, and Biosynthesis of ABA

To further understand the protective role of Si and B, we measured the expression pattern of genes in leaves and roots related to Si (silicon efflux transporter; *Lsi2*) and Al^3+^ transport (*Phoenix dactylifera aluminum-activated malate transporter 1-like; ALMT1*, and *ALMT2*), ATPase H^+^ pump (*Phoenix dactylifera plasma membrane ATPase; PMMA1*, and *PMMA3*), and the biosynthesis of ABA (*abscisic acid receptor PYL4-like; PYL4, abscisic acid receptor PYR1; PYR1, CAT, Cyt-Cu/Zn SOD*, *9-cis-epoxycarotenoid dioxygenase; NCED1, and* *9-cis-epoxycarotenoid dioxygenase; NCED6*) at the molecular level by qRT-PCR.

**Figure 6:**
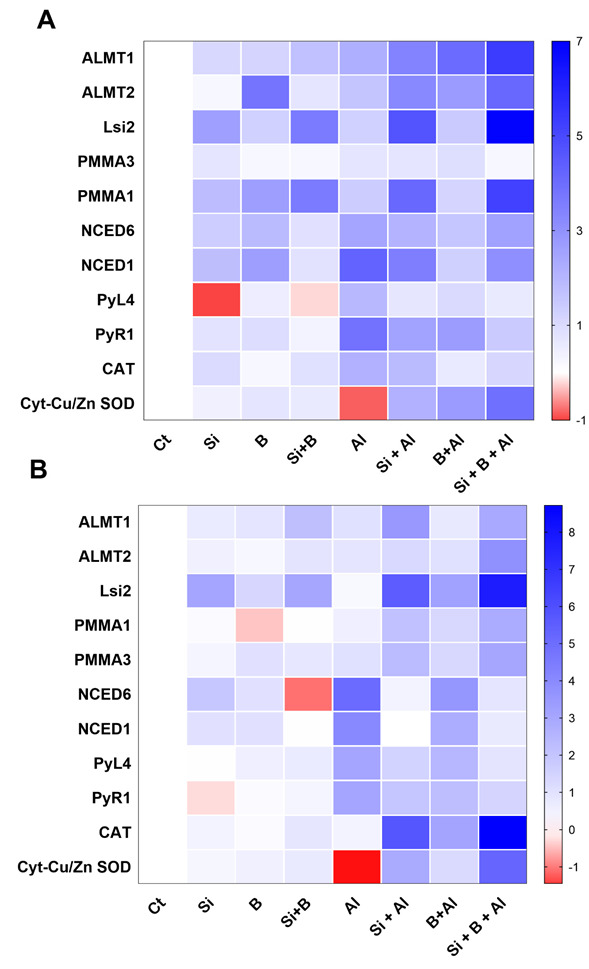
The sole and in combination effect of Si and B on the regulation of gene expressions in date palm (**A**) shoots and (**B**) roots under control and Al-induced stress conditions. The red color represents a lower relative transcript accumulation concerning control, and the blue color represents a higher transcript accumulation.

As shown in Figure 6A, the application of Si, B, and their combination induced some vital and significant variations in the expression levels of most of the evaluated genes of date palm shoots compared to control plants under Al^3+^ stress. The treatment of sole Si, B, and their combination significantly induced the expression of *ALMT1* and *ALMT2* as compared to sole Al3+-treated plants under aluminum stress. Likewise, the *Lsi2* gene was significantly upregulated in all the seedlings treated with Si under conditions and Al^3+^ stress conditions compared to non-Si treated plant shoots. However, the expression of *Lsi2* gene was significantly enhanced under the aluminum stress of Si-treated plants compared to Si-treated plants under control conditions. Additionally, the transcript accumulation of *PPMA3* was significantly downregulated by the combination of Si + B under control and Al^3+^ stress conditions compared to other treatments. However, the combination of Si + B and sole Si significantly induced the transcript accumulation of *PPMA1* as compared to sole Al^3+^ treated and B treated plants under aluminum stress.

Similarly, no significant differences in the expression pattern of *NCED6* were induced under aluminum stress by sole Si, B, and their combination compared to sole Al^3+^ treatment, whereas the significantly enhanced expression of *NCED1* was detected in sole Al^3+^ treatment, and the lowest was noticed in sole Bi-treated plants shoots. The over expression of *PYL4* and *PYR1* was detected in sole Al3+-treated plants, while significant downregulation was observed in Si + B-treated plants. Furthermore, *Cyt-Cu/Zn SOD* expression was detected significantly lower in sole Al^3+^ treatment, while the combination of Si + B presented a significantly enhanced expression level under aluminum stress.

Likewise, the expression of the genes mentioned earlier was also determined in the roots of date palm seedlings (Figure 6B). Interestingly, plants treated with Si, B, and their combination presented a different expression pattern of *Lsi2*, *NCED6*, and *PYR1* compared to control plants under the no-Al^3+^ stress. We found that *ALMT1* and *ALMT2* genes were upregulated in Si + B-treated seedlings under Al^3+^ stress compared to sole Al3+-treated plants and sole B-treated plants. Furthermore, the *Lsi2* gene revealed a higher expression level in all Si-treated plants, and sole Al3+-treated plants presented significantly lower expression under aluminum stress. Moreover, *PPMA1*, *PPMA3, CAT*, and *Cyt-Cu/Zn SOD* expression level was detected to be extremely lower in sole Al3+-treated plants, while a significantly enhanced expression level was exhibited in combined Si + B-treated plants under aluminum stress. The transcript accumulation of *NCED1, NCED6*, *PYL4*, and *PYR1* were significantly upregulated in sole Al3+-treated plants and significantly down-regulated in combined Si + B-treated plant roots.

## 4. Discussion

Excessive metal contamination has destructive impacts on plant growth, physiology, metabolic processes, and productivity. As a toxic metal, aluminum poses a severe threat to agricultural plants and field soil due to excessive acidification by intensive anthropogenic activities and global environmental changes [54]. From past research, it has become evident that the exogenous application of Si can lead to the amelioration of Al toxicity in plants and soil by modulating the soil pH redox potential, enzymatic activities, its accumulation, transportation, and translocation in plants at a cellular level, and the modulation of root-hair morphology, nutrient uptake, and transportation systems [55]. Owing to its monomeric or monosilicic nature, Si is easily absorbed by roots in soil to augment growth attributes and decrease metal accumulation and translocation from roots to shoots, regulating the antioxidant system, metal chelation, and sequestration and inducing the silicon transporters system for alleviating metals stress [56]. However, information is lacking regarding the mitigation and the underlying mechanism of the toxic effects of Al on date palm via the exogenous treatment of Si, particularly in terms of co-synergism with the B application. The findings of the current study illustrate the detrimental effects of Al stress on date palm growth parameters, including shoot and root length, fresh and dry biomass, chlorophyll, and carotenoid contents.

Conversely, the exogenous application of Si to date palm significantly ameliorated the adverse impacts of Al toxicity by enhancing plant growth attributes. The impact of exogenous Si coupled with B was more significant in lessening Al stress in date palm by exhibiting extremely significant growth attributes compared to individual Si and B treatment. The enhanced growth of date palm under Al stress could be attributed to the improved production of chlorophyll (*a* and *b*) content and carotenoid levels. Previously, Chen et al. [43] reported that boron and silicon interaction augmented chlorophyll and carotenoids levels in rice and led to improved growth attributes under heavy metals stress. Similarly, an enhanced level of chlorophyll and carotenoids with a significant growth rate was also observed by applying B to sunflower, wheat, and cotton exposed to drought and salinity, respectively [57,58,59].

Moreover, the inhibition of photosynthetic pigments by metals toxicity, including Al stress, is also reported to reduce root and shoot growth in *Azolla* and *Secale* cereal plants due to the excessive generation of ROS and interfering in essential nutrients uptake required for chlorophyll synthesis [60,61]. In the current study, the same phenomenon of the degradation photosynthetic pigments was detected in sole Al3+-treated plants, which significantly reduced growth attributes in date palm. However, compared with sole Si, B, and Al^3+^ treatments, the combined application of Si + B demonstrated the significant mitigation of Al-induced stress and revealed higher photosynthetic pigments for maintaining a better growth rate.

The uptake of Al and its translocation along with Si and B from roots to shoots was scrutinized to gain a better insight into the ameliorating effect of Si coupled with B under Al toxicity in date palm. A significant reduction in Al uptake by roots and its translocation to shoots was observed in sole Si and B treatment compared to sole Al^3+^ treatment. However, the effect of combined Si + B on inhibiting Al uptake and its translocation was significantly higher than in sole treatments. In parallel, the enhanced deposition of Si in roots and its translocation to the upper part was detected in Si-treated plants, which is known for blocking the passage of lethal cell-degrading ions, such as Al, through forming complexes and the precipitation of metal ions as a co factor [52,62]. Moreover, inhibiting the uptake and translocation of Al by coupled application Si + B could also be ascribed to the significant regulation of organic acids in roots and shoots. It can be assumed from the regulation of organic acids that date palm under Si + B application has adopted an apoplasmic mechanism for restricting Al entry to the cell wall. Previously, for the reduction and inhibition of Al toxicity, the induction of organic acids into the apoplastic spaces for encountering the binding of Al to pectin is reported as the best mechanism of Al tolerance in plants [63,64]. In the current study, the synergistic effect of Si + B significantly enhanced the regulation of aluminum-activated malate transporters genes (*ALMT1* and *ALMT2*), which plants frequently use to exclude Al from the root [65,66]. The significant activation of *ALMTs* by combined Si and B was also evident from the enhanced production of malic acids and citric acids in roots could be the possible justification for the exclusion of Al from roots cells via encountering the attachment to pectin and thereby lead to the alleviation of the generated toxicity [64,67].

Additionally, the effect of Si, when coupled with B, was more predominant in ameliorating Al toxicity via the upregulation of *ALMTs* transporters and the activity of silicon efflux transporter (*Lsi2*) genes, suggesting the intense requirement of Si by tissues for encountering Al toxicity. In line with the current findings, Pontigo et al. [9] reported the upregulation of Silicon efflux transporter genes and silicon uptake for inhibiting Al uptake in ryegrass. Similarly, the overexpression of the *ALMT1* gene in blackgram induced organic acid including malate exudation and led to reduced Al^3+^ uptake and better growth attributes and photosynthetic efficiency. The current findings indicated the Al-mitigating effects of combined Si and B application to date palm via inducing organic acid regulation and silicon efflux transporters to reduce the uptake and accumulation for better plant growth.

Furthermore, plasma membrane ATPases regulation is vital for modulating the plant physiological processes required for resisting or tolerating metals toxicity, including Al stress in plants. The alteration of plasma membrane H^+^-ATPase-inducing H^+^ influx is interlinked with the maintaining cytosolic pH, the electrochemical gradient of the plasma membrane, and Al-induced citrate efflux [66]. The current study depicted a strong effect of combined Si and B application on inducing *PMMA1* and *PMMA3* expression under Al stress, indicating that the higher citric acid production in date palm had subsequently activated plasma membrane H^+^-ATPase activity for counteracting Al-toxicity. The current results also coincide with Dawood et al. [68], who found suppressed proton adenosine triphosphatase (H^+^-ATPase) activity barely under aluminum toxicity, leading to the higher accumulation of Al and the inhibition of growth in plants. Moreover, B is also reported to induce root surface alkalization to reduce Al uptake and accumulation in apical zones through the induced auxin efflux transporter and subsequently trigger the downstream regulation of plasma membrane-H^+^-ATPase [5,69]. The synergistic effects of Si and B highlight their impacts on coping with Al toxicity in date palm via the regulation of plasma membrane H^+^-ATPase activity and aluminum-activated malate transporter genes via the exudation of organic acids.

Al toxicity is also considered lethal for triggering lipid membrane disruption and producing ROS [70]. The current findings indicated significant lipid peroxidation activities in higher MDA production and O_2_^•−^ accumulation in the roots and shoots of sole Al3+-treated seedlings. Conversely, the combined application of Si + B demonstrated the significant mitigation of O_2_^•−^ and MDA by triggering the upregulation of the antioxidant system to combat Al-induced toxicity. The result suggests that the application of Si + B jointly aids plant antioxidative defense systems and provides Al-tolerance to date palm by upregulating the expression of *Cyt-Cu/Zn SOD* and *CAT*, thereby inhibiting the ROS indicators i.e., O_2_^•−^, and MDA. The in-tandem activation of *cyt-Cu/Zn SOD* and APX activities by the combined treatment of Si and B is an indicator of conferring resistance to Al toxicity in date palm. In line with our findings, the combined regulation of *Cu/Zn SOD* and APX significantly improved the tolerance level of transgenic cassava plants to abiotic stress [71]. The activation of the antioxidant system for the amelioration of ROS could also be ascribed to the modulation of the exudation of organic acids such as citric acids, malic acids, and acetic acids via altering the *plasma membrane ATPase* and *aluminium-activated malate transporter* gene regulation by combined Si and B effects. In agreement with this, Tahjib-Ul-Arif et al. [72], Zhang et al. [73], and Bilal et al. [70] also revealed the role of the exogenous application and exudation of organic acids, including citric acids, in providing resistance to plants to combat excessive ROS production under abiotic including metals stress. However, further investigation at the transcriptomic level is required to explore the precise mode of action and insights mechanism of the Si-B regulated antioxidant system of the date palm under Al-toxicity.

Stress-signaling endogenous hormonal modulation and crosstalk are vital for regulating plant metabolism under hostile conditions [74], as is the ability of Si to form complexes with stress-inducing heavy metals and inhibit their uptake and translocation, consequently influencing plant defense-related hormonal regulation [28,56,75,76]. The biosynthesis of ABA in plants for imparting tolerance to stresses, including drought, and metals, is of key importance. The role of ABA signaling in activating a plant defense system to varying levels of Al stress has been reported previously [77]. The current study’s findings demonstrated that the synergistic impacts of Si and B resulted in a significant reduction in the ABA level under Al-stress in date palm. Such a reduction in ABA accumulation under Al toxicity could be ascribed to the generation of less oxidative stress, i.e., ROS elimination and the corresponding downregulation of the abscisic acid receptor *PYL4* and *PYR1 9-cis-epoxycarotenoid dioxygenase 1* and *6* genes. The current findings highlight the Al protective role of synergistic Si and B application by reducing the hazardous effects of Al toxicity for date palm. Consequently, ABA signaling and accumulation were not activated. However, in the case of sole Al^3+^ plants, the toxicity of Al led to the significantly higher regulation of *NCED* genes along with *PYL4* and *PYR1*, leading to the enhanced biosynthesis of ABA to activate the plant defense system to cope with the generated stress. These findings indicate that the combined effect of Si + B induced the ABA-independent pathway in date palm to withstand Al-induced toxicity [78,79].

Moreover, the higher uptake of silicon in combined application compared to the sole application of Si and B could also be attributed to the reduced ABA activities ( Tripathi et al. [80] have also described the effect of silicon uptake on reducing ABA activities under abiotic stress in plants). Moreover, the crosstalk of endogenous SA with ABA is crucial for regulating plant growth and detoxifying excessive ROS under abiotic stresses [81]. The current findings describe the antagonistic trend of SA accumulation compared to reduced ABA accumulation under Al toxicity in combined Si- and B-treated plants. The increase in SA accumulation under combined treatment is reported to contribute to the formation of a protective layer on the leaves; the improvement of the antioxidant system; and the reduction of the lipid peroxidation by the regulation of phenolic compounds, flavonoids, and PAL activities [82]. Contrarily to the current increase of SA content by the synergistic effect of Si and B, the authors of [83] described a significant reduction in SA accumulation under heavy metal toxicity by exogenous Si treatment in rice plants. Such higher regulation of SA accumulation, particularly in roots by the synergistic effect of Si and B, could be the reason for the excessive exudation of organic acids such as citric acid and indicate SA involvement in encountering Al toxicity and thereby enhancing plant tolerance and growth attributes to the stress condition. In concurring with the current findings, the higher regulation of SA is also reported for mediating Al tolerance to plants via modulating citrate efflux from roots and initiating the SA signal transduction pathway in *Cassia tora* L. plants [84]. The effect of B uptake and accumulation under the combined treatment of Si and B in the control and the Al stress condition could also be ascribed to the higher endogenous SA accumulation in date palm as the uptake and translocation of B are reported to enhance endogenous SA content in plants for modulating plant growth and defense systems under hostile conditions [85]. The current findings demonstrated the ameliorative effects of the synergistic application of Si and B for modulating regulatory hormonal systems to combat Al-toxicity and improve plant physiological processes.

## 5. Conclusions

The current study revealed the significant growth and biomass enhancement of date palm under toxic effects of Al by the synergistic impacts of Si and B. The synergism of Si and B led to the enhancement of Si accumulation for boosting plant growth and modulating physiological and biochemical alterations in date palm. The synergistic performance of Si and B was characterized by reducing Al^3+^ uptake and translocation in date palm. Such a response was associated with the differential modulation of *ALMTs* and *PMMAs*, thereby inducing the exudation of organic acids such as malic acid and citric acids for counteracting Al3+-induced toxicity in soil. In parallel, Al^3+^ induced oxidative stress was alleviated by lowering MDA and O_2_^•–^ via the enhanced regulation of *CAT*; *Cyt-Cu/Zn SOD*; and CAT, POD, and APX activities. Likewise, the hazardous effect of Al toxicity was alleviated by combined Si and B application through the downregulation of the abscisic acid receptors *PYL4* and *PYR1* and the *NCED1* and *NCED 6* genes, resulting in the lower accumulation of endogenous ABA and the marked regulation of SA. The combined application of Si and B convened significant tolerance in date palm to combat the Al stress-related damages by maintaining plant growth attributes and the organic acids’ exudation and regulation of the oxidative metabolism for triggering hormonal cross talk. Furthermore, the investigation is required at a transcriptomic level to dissect the underlying mechanism of Si and B interaction for ameliorating Al-induced damages in date palm.

## Figures and Tables

**Table 1:** The exogenous application of Si and B alone and in combination with date palm.

Treatments	Shoot Length (cm)	Root Length (cm)	Shoot Dry Weight (mg)	Root dry Weight (mg)	Chl *a* (mg g^−1^ FW)	Chl *b* (mg g^−1^ FW)	Carot (mg g^−1^ FW)
Ct	20.0 ± 1.51 ^e^	6.6 ± 0.69 ^b^	1371.7 ± 71.69 ^d^	1144.0 ±108.75 ^b^	61.6 ± 5.8 ^a^	113.6 ± 11.4 ^e^	109.5 ± 8.2 ^f^
Si	28.0 ± 1.15 ^b^	8.4 ± 0.69 ^a^	2090.8 ±107.78 ^b^	1390.6 ± 104.47 ^a^	65.1 ± 4.6 ^a^	174.4 ± 12.6 ^b^	151.2 ± 6.6 ^d^
B	26.6 ± 1.03 ^c^	8.7 ± 1.05 ^a^	1936.6 ±103.94 ^b^	1309.6 ± 55.23 ^a^	66.8 ± 1.6 ^a^	183.1 ± 11.9 ^b^	168.2 ± 6.8 ^c^
Si + B	31.1 ± 1.75 ^a^	9.6 ± 0.84 ^a^	2442.2 ±116.21 ^a^	1438.8 ± 126.56 ^a^	65.5 ± 6.3 ^a^	216.0 ± 18.1 ^a^	191.0 ± 6.1 ^b^
Al^3+^	15.4 ± 1.95 ^f^	4.9 ± 1.19 ^c^	980.0 ± 72.94 ^e^	784.00 ± 62.44 ^c^	33.1 ± 5.2 ^b^	57.2 ± 10.4 ^f^	83.6 ± 6.5 ^g^
Si + Al^3+^	24.1 ± 1.07 ^cd^	7.7 ± 1.15 ^a^	1597.8 ±123.05 ^c^	1094.8 ± 48.59 ^b^	62.0 ± 2.2 ^a^	149.8 ± 14.3 ^cd^	139.0 ± 5.8 ^e^
B + Al^3+^	22.5 ± 1.14 ^d^	8.4 ± 1.26 ^a^	1627.0 ±105.03 ^c^	968.20 ± 34.13 ^b^	60.0 ± 5.8 ^a^	137.0 ± 6.97 ^d^	155.8 ± 4.9 ^d^
Si + B + Al^3+^	26.0 ± 1.53 ^bc^	9.2 ± 1.13 ^a^	2003.6 ± 110.59 ^b^	1086.2 ± 124.86 ^b^	59.5 ± 11.0 ^a^	156.6 ± 11.9 ^cb^	204.6 ± 8.7 ^a^

The growth parameters of date palm seedlings under normal and aluminum stress conditions such as shoot and root length, shoot and dry weight, chlorophyll (Chl *a*), (Chl *b*), and carotenoids (Carot). The different letter(s) in each row indicate a significant difference (*p* < 0.05) among all treatments by DMRT.

**Table 2:** The accumulation and uptake of B, Si, and Al^+^ by the roots and shoots of date palm under aluminum stress.

Treatment	B µg g^−1^		Si µg g^−1^		Al µg g^−1^	
	Leaves	Root	Leaves	Root	Leaves	Root
Ct	45.04 ± 3.64 ^e^	28.63 ± 3.83 ^cd^	381.89 ± 10.31 ^e^	306.02 ± 11.3 ^f^	18.44 ± 2.64 ^c^	127.52 ± 7.1 ^ef^
Si	41.23 ± 4.30 ^ef^	25.06 ± 1.81 ^d^	673.08 ± 21.32 ^a^	509.36 ± 21.2 ^a^	22.91 ± 2.52 ^c^	114.49 ± 6.23 ^fg^
B	75.57 ± 4.57 ^b^	50.36 ± 2.96 ^a^	433.53 ± 18.12 ^d^	296.52 ± 14.5 ^f^	42.38 ± 3.29 ^c^	98.32 ± 6.62 ^g^
Si + B	36.25 ± 3.14 ^f^	39.27 ± 3.04 ^b^	513.36 ± 25.34 ^c^	456.72 ± 17.2 ^b^	31.45 ± 2.72 ^b^	144.89 ± 8.33 ^e^
Al	47.94 ± 3.04 ^e^	23.64 ± 2.71 ^d^	346.57 ± 14.55 ^f^	229.91 ± 21.7 ^g^	116.86 ± 8.31 ^a^	541.29 ± 29.1 ^a^
Si + Al	58.61 ± 3.51 ^d^	32.91 ± 3.31 ^c^	572.89 ± 22.32 ^b^	424.52 ± 12.5 ^c^	68.23 ± 3.556 ^b^	323.74 ± 23.02 ^b^
B + Al	89.23 ± 5.06 ^a^	49.43 ± 3.79 ^a^	403.01 ± 19.33 ^e^	331.97 ± 11.4 ^e^	83.95 ± 4.63 ^b^	276.71 ± 16.74 ^c^
Si + B + Al	68.16 ± 4.63 ^c^	49.95 ± 5.32 ^a^	522.90 ± 16.52 ^c^	379.67 ± 18.1 ^d^	50.60 ± 3.12 ^b^	218.05 ± 11.72 ^d^

Date palm seedlings grown under normal and Al stress conditions with/without exogenously applied Si, B, and Si + B. The different letter(s) in each row indicate a significant difference (*p* < 0.05) among all treatments by DMRT.

## Data Availability

Data is contained within the article or Supplementary Material.

## Supplementary Materials

The following supporting information can be downloaded at: https://www.mdpi.com/article/10.3390/antiox11061063/s1, Supplementary file S1: ABA, SA analysis methodology; Table S1: The gene name, gene description, product size, reference number and oligonucleotide sequences used for qRT PCR.
