# Supplementary material for: Silicon- and Boron-Induced Physio-Biochemical Alteration and Organic Acid Regulation Mitigates Aluminum Phytotoxicity in Date Palm Seedlings
Source: Antioxidants (Basel). 2022 May 27;11(6):1063. doi: 10.3390/antiox11061063 (PMC9219922; doi:10.3390/antiox11061063)
Supplement: Supplementary file 1 [file antioxidants-11-01063-s001.zip › Supplementry File S 1.pdf]

ABA ANALYSIS

1. Take freeze dry sample and weight the sample 0.2-0.5 gram.
2. Label the flask and add 10 ml of ISO-Propanol to 95 % Iso-Propanol: 5% Acetic Acid and cover the flask with Aluminum foil and keep on shaker for 20 mints at speed of 130-140 rpm.
3. Filter the sample through Vacuum Filtration.
4. Wash each flask with Iso-Propanol 5ml 4 times and wait to filter by itself.
5. After self-filtration filter through vacuum filtration.
6. Transfer the solution from filtration flask to RBF and add 0.5 ml standard of ABA to each flask and wait for some time.
7. After adding standard, evaporate the sample through Rotary Evaporator but leave some small amount max 2 ml.
8. After drying Add 2ml 1N NaoH to RBF and Sonicate it and transfer to long test tube again add 3 NaoH and do the same process.
9. Adjust the pH 12.5-13, during pH setting put magnet for shaking
   (Add NaoH/ HCL for pH up and Down)
10. After adjusting H vertex the test tube.
11. Add 4 ml Dichloro-Methane to the respective empty RBF Sonicate it and transfer to their respective test tube. (4,3,3 ml each time)
12. After adding solution from RBF to test tube vertex the tube and wait for some time to make layers.
13. After formation of layers remove the down layer to another test tube for Discard, we need Upper Layer. At the same time add 4 ml DICHLORO-METHANE to the respective empty RBF, Sonicate it and transfer to their respective test tube, vertex the tube and wait for some time to make layers and repeat the process 2 times (4,3,3 ml).
14. We need upper layer. After removing the down layer adjust the pH of upper layer from 2.5-3.5.
15. After adjusting the pH again do the solvent-solvent partition with ETHYL-ACETATE by adding 4 ml into the test tube, 3 times.
16. After adding ETHYL-ACETATE vertex and remove the upper layer to new RBF, 3 times.
17. After finishing the above steps dry the solution completely in RBF through R.Evaporator.

18. Take 1 gram of PvPP (POLYVINY PYRILLODONE) to new flask.
19. Wash the dry RBF 3 times with 4 ml of Phosphate buffer having pH-8 and Sonicate and transfer to their respective label flask having 1 g of PvPP. (4,3,3 ml)
20. Put the flask in shaker for 1-1.30 hours.
21. After shaking, filter the solution by vacuum filter by putting test tube inside the vacuum flask.

    Clean the flask with 5 ml PHOSPHATE BUFFER 2 times.
22. After filtration adjust the pH 2.5-3.5.
23. Solvent-solvent separation. After adjusting the pH again repeat solvent-solvent separation process by adding 4 ml ETHYL ACETATE and vertex the test tube.

    3 time by removing the upper layer of solution in test tube. The upper layer will be further processed. 4+3+3 total 3 times.
24. Add 2. 5 ml of 2.5 pH DDW to ethyl acetate and vertex and discard the lower portion (which is water) repeat it for 2-3 times.
25. Transfer the solution to RBF and transfer to rotary evaporator for drying the solution at 40-50 °c but leave 1 ml.
26. Transfer the 1 ml to 1 ml vial and keep it on N-dryer.
27. After drying add 1 ml (100%) ETHYL ACETATE to RBF 2 times and Sonicate and transfer to 1 ml vial and dry the vial completely by N-dryer.
28. Put 60 µ L DIAZOMETHANE into vial and vertex it. After 30 mints dry it and again add 60 µL DIAZOMETHANE and cover with ALUMINUM FOIL and after 1 hour it will be ready for another step.
29. After 1 hour dry the vial on N-dryer and add 20 µ L DICHLOROMETHANE (CH2CL2) and then vertex it and will be ready for injection.

SA extraction

1- Take 0.3g of the plant fresh/freez dry sample in large test tube.
2- Add 2ml of 90% methanol to each tube and keep on sonicate for 20-25 mint.
3- Transfer the salution to centrifuage tube.
4- Wash the tube with 0.5ml of 90% methanol and transfer to same centrifuge tube (2 time).
5- Centrifuage at 12000rpm for 15 mints.
6- Transfer the supernatant to new test tube.
7- Add 2.5 ml of 100% methanol to the same centrifuge tube.
8- Centrifuage at 1200g for 15 mints.
9- Transfer the supernatant to same test tube.
10- Divided the salution in to 2 test tube with equal amount and vacuum dry the sample using savant vacuum system for 2-3h.
11- After drying add 2ml of 5% TCA to each pellet and sonicate for 10 mints, and transfer the salution to centrifuage tubes. (wash the same dry test tube with 0.5ml of 5% TCA and transfer to the same centrifuage tube).
12- Centrifuage it for 10 mints at 10000rpm
13- Transfer the supernatant to new test tube and add Ethyle acetate: cyclopentane: isopropanol 49.5%: 49.5%: 1%, and vertex.
14- Removed the upper layer to 5ml vail and keep on N-dry.
15- After dry add 1ml 100% methanol, sonicate and vertex it, after that filter through syreng filter in an new tube. Ready for injecting in HPLC
