# Supplementary material for: Silicon- and Boron-Induced Physio-Biochemical Alteration and Organic Acid Regulation Mitigates Aluminum Phytotoxicity in Date Palm Seedlings
Source: Antioxidants (Basel). 2022 May 27;11(6):1063. doi: 10.3390/antiox11061063 (PMC9219922; doi:10.3390/antiox11061063)
Supplement: Supplementary file 1 [file antioxidants-11-01063-s001.zip › Table S1.pdf]

Table S1. The gene name, gene description, product size, reference number and oligonucleotide sequences used for qRT-PCR.

Gene name	Description	Primer sequence (5′-3′)	Size (bp)	Accession
Lsi2	Silicon efflux transporter	F: GCTTCTTCCTGCGATGCTTG<br>R: CGTCTTTGGCTGCTTGAACC	116	XM_026801795.1
ALMT1	Phoenix dactylifera aluminum-activated malate transporter 1-like	F: CAGCATCCTTGCTCTCACAA<br>R: TATGAAGGGTCTCGCAATCC	105	XM_008787669.1
ALMT2	Phoenix dactylifera aluminum-activated malate transporter 1-like	F: GCTCTTTGGTGGCTCTTTTG<br>R: CGAGAATATGTGGCCATTCC	103	XM_026805000.1
PPMA1	Phoenix dactylifera plasma membrane ATPase 1	F: GTGGGATGACTGGAGATGGAGT<br>R: TGCACGGCTAGTTAAGACAGCA	120	XM_008800569.3
PPMA3	Phoenix dactylifera plasma membrane ATPase 3	F: CAGTCGTGCCATTTTTCAGA<br>R: AAAATCTCAGCCAGCTTCCA	118	XM_008798430.2
PYL4	Abscisic acid receptor PYL4-like	F: CGTCGAGTCCTACGTTGTCG<br>R: GCCAGGTTCTCGGAGGTATG	120	XM_008801643.3
PYR1	Abscisic acid receptor PYR1	F: ACGGTGGTGCTGGAATCGTA<br>R: GAGGCGAGCTTCTGGAGGTT	110	NW_008246541.1
ZEP	Zeaxanthin epoxidase	F: CCACTCTTTCCTCGCCAATC<br>R: GAATCTGGCAATGGGCTTTC	120	XM_008794293.2
Cyt-Cu/Zn SOD	superoxide dismutase [Cu-Zn]-like	F: AAGCCTCTCTGGCCTCGAA<br>R: CACCGAGGGCATGAACATG	110	XM_008791563.3
NCED1	9-cis-epoxycarotenoid dioxygenase	F: GCCTCCTCGCACCACTATTC<br>R: CGGGTCGATGAAGTTGTTGA	120	XM_008799733.3
NCED6	9-cis-epoxycarotenoid dioxygenase	F: GCCTCCTCGCACCACTATTC<br>R: CGGGTCGATGAAGTTGTTGA	120	XM_008799733.3
Act	Housekeeping gene actin	F: TCAATGTGCCTGCCATGTATGT<br>R: GCGGCCGCTAGCATAGAG	62	XM_008778129
